# A Double Edged Sword Role of Interleukin-22 in Wound Healing and Tissue Regeneration

**DOI:** 10.3389/fimmu.2020.02148

**Published:** 2020-09-17

**Authors:** Tanzeela Arshad, Fizzah Mansur, Richard Palek, Sobia Manzoor, Vaclav Liska

**Affiliations:** ^1^Molecular Virology and Immunology Research Group, Atta-ur-Rahman School of Applied Bio-Sciences, National University of Sciences and Technology, Islamabad, Pakistan; ^2^Department of Surgery, Faculty of Medicine in Pilsen, Charles University, Pilsen, Czechia; ^3^Laboratory of Cancer Treatment and Tissue Regeneration, Biomedical Centre, Faculty of Medicine in Pilsen, Charles University, Prague, Czechia

**Keywords:** IL22-producing cells, wound healing—physiopathology, tissue regeneration, carcinogenesis and metastasis, innate immune response and inflammation

## Abstract

Wound healing and tissue regeneration is an intricate biological process that involves repair of cellular damage and maintenance of tissue integrity. Cascades involved in wound healing and tissue regeneration highly overlap with cancer causing pathways. Usually, subsequent tissue damage events include release of a number of cytokines to accomplish post-trauma restoration. IL-22 is one of the cytokines that are immediately produced to initiate immune response against several tissue impairments. IL-22 is a fundamental mediator in inflammation, mucous production, protective role against pathogens, wound healing, and tissue regeneration. However, accumulating evidence suggests pivotal role of IL-22 in instigation of various cancers due to its pro-inflammatory and tissue repairing activity. In this review, we summarize how healing effects of IL-22, when executed in an uncontrollable fashion can lead to carcinogenesis.

## Introduction

IL-22 is a key signaling molecule that plays a significant role in number of essential physiological processes ranging from innate immune responses to tissue regeneration. Up-regulation and down-regulation of IL-22 generate several consequences that define its biological and pathological activities (1, 2). Owing to pivotal role of IL-22 in normal biological functions, any impairment in its activity can lead to chronic inflammatory diseases, disturbed wound healing, infections, and cancers (3, 4). This dual role highlights the therapeutic prospective of modulating the cytokine network to achieve tumor prevention and treatment.

## Aims and Objectives

IL-22 is implicated in a large variety of functions in the body, given its involvement in signaling pathways. IL-22 has been shown to be a critical signaling molecule in regenerative processes and on the other hand, certain pathological conditions in several organs depending on its environmental factors, cytokine milieu and context. Because of its extensive involvement in regeneration, host defense and pathological conditions, IL-22 has become an important target for clinical and therapeutic development. Therefore, it is imperative to understand the detailed pathways of IL-22 that can convert a normal physiological process into a pathological environment.

In this review, we have, in detail, discussed the present state of knowledge about the biology of IL-22, its sources and targets, and its role in regeneration that could, in uncontrolled conditions, lead to tissue pathology or carcinogenesis.

## IL-22 Biochemical Properties

IL-22, a novel protein, was discovered for the first time by Dumoutier et al. in the year 2000 (5). Initially, IL-22 was called Interleukin-10-related T cell-derived inducible factor due to its significant structural resemblance (homology of 22%) to mouse interleukin-10 (5). Further characterization and cloning of IL-22 revealed the structure of this cytokine to be alpha helical, comprising of 179 amino acids containing six α-helices which are usually known as A to F helices. A monomeric bundle like conformation is formed by these helices, which bind together in an anti-parallel fashion (6–8). IL-22 is a class II cytokine and has been categorized in the IL-10 family of cytokines due to its biochemical and functional characteristics, along with other interleukins including IL-10, IL-28, IL-26, IL-29, IL-24, and IL-20 (9). In humans, chromosome 12 contains the gene responsible for encoding IL-22. It is present upstream of the genetic loci for IFN-γ (interferon gamma) and IL-26 (10). The secreted form of IL-22 generally consists of 146 amino acids (5, 8, 11). Characteristically, the functional form of IL-22 exists as a monomer, but on the other hand, it also has the ability to form dimers and tetramers, which serve as non-functional storage forms (12).

### IL-22 Receptors

For elicitation of its downstream effector functions, IL-22 employs its receptor complex which functions as a heterodimeric complex and is composed of two receptors; IL-22R1 and IL-10R2 (6, 13, 14). This receptor complex is able to further activate a multitude of downstream signaling cascades (14). IL-22R1 and IL-10R2 have been categorized in the class II cytokine receptor family (13). The gene for IL-22R1 is present on chromosome 1p36.11 in close proximity to the IL-28RA gene, and the gene responsible for encoding IL-10R2 is found on the chromosome 21q22.11, close to the IFNARA, IFNARB, and IFNGRB genes (15, 16). A very high binding affinity has been reported between IL-22 and IL-22R1, whereas it has been observed that IL-22 shows a low affinity for IL-10R2 (17, 18). When IL-22 encounters IL-22R1, it binds to the receptor owing to its high binding affinity, which in-turn results in the occurrence of a conformational modification of the cytokine. This modification causes an increase in the affinity of IL-22 to bind to IL-10R2 thereby allowing IL-22 and IL-22R1 complex to bind to IL-10R2 (7, 19). IL-22 receptor complex is transmembrane complex associated with Janus kinase (JAK) and Tyrosine Kinases (TYK). When IL-22 binds with its receptor complex, it triggers a number of downstream cascades including JAK, TYK2 phosphorylation which further phosphorylates and activates STAT-3 (signal transducer and activator of transcription 3) which is responsible for a wide array of downstream effects. Some other molecules including p38, ERK (extracellular signal-regulated kinases), JNK (c-Jun N-terminal kinases), PI3K (phosphoinositide 3-kinases) are also activated downstream of IL-22 binding, thereby mediating IL-22 in eliciting its effector functions (1, 20, 21).

### IL-22 Binding Protein

Another receptor for IL-22, namely IL-22 binding protein (IL-22BP) exists in the form of a soluble receptor. It has been reported that IL-22 has a very high affinity for binding to IL-22BP (22, 23). IL-22BP acts as a natural antagonist of IL-22 since it binds to IL-22 at specific sites that are also utilized by IL-22R1, thereby blocking the binding of IL-22 to its membrane bound receptor (24). Owing to a stable association with IL-22, IL-22BP sequesters IL-22 away from IL-22R1 thereby precluding its downstream signaling activation (25). IL-22BP has been reported to be expressed in numerous tissues of the body, including lungs, skin, lymphatic tissue, placental tissue, breast, and gastrointestinal tract. Dendritic cells play an important role in producing IL-22BP, along with epithelial cells, macrophages, and eosinophils (26).

## IL-22 Sources and Targets

**Figure 1:**
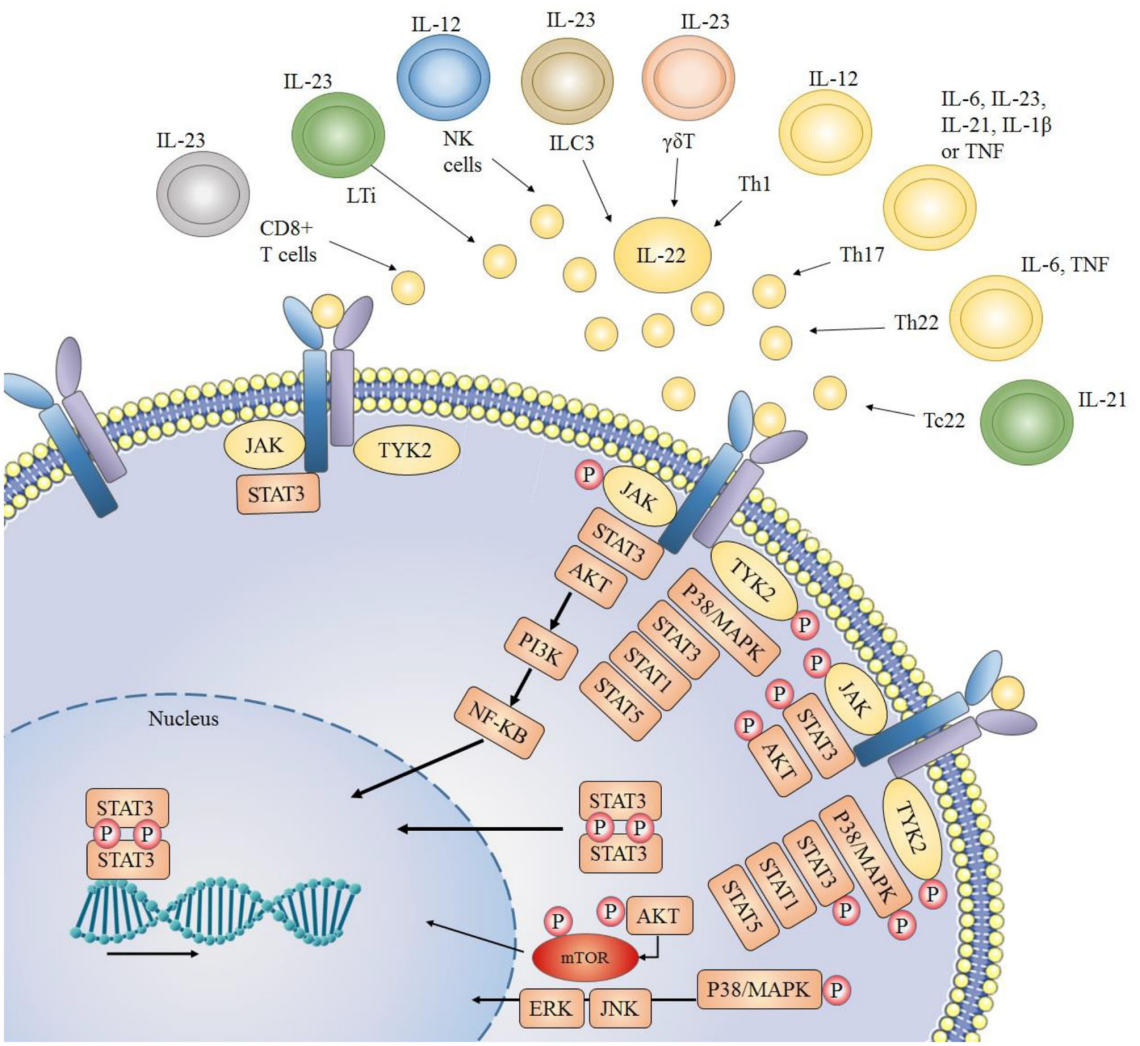
Sources of IL-22 and downstream signaling pathways: IL-22 is mainly secreted by immune cells. JAK1 (Janus kinase 1) and TYK2 (Tyrosine kinase 2) molecules are associated with the cytoplasmic region of IL-22 receptor. When the IL-22 receptor is activated (due to a complex formation between its two heterodimeric receptors), JAK1 and TYK2 are phosphorylated, which further phosphorylate STAT (signal transducer and activator of transcription) molecules thus resulting in dimerization and consequent translocation of STATs to the nucleus. Usually STAT3 molecules play key role, but STAT1 and STAT5 may also be involved. Other than JAK/STAT activation and downstream signaling, IL-22 also activates PI3K/AKT (phosphoinositide 3-kinases/protein kinase B) and MAPK (mitogen-activated protein kinase) pathways through JAK and TYK2 molecules.

T lymphocytes specifically CD4+ T cells or T helper (Th) cells act as the primary sources of IL-22 production (27). A unidirectional flow of cytokine signaling can be seen in the case of IL-22, as immune cells act as the main source of secretion for IL-22, while its main targets include non-hematopoietic epithelial cells. For this reason, IL-22 is considered as an essential factor of immune-epithelial cross talk. Th1 and Th17 cells are major producers of IL-22 [Figure 1; (28–30)]. IL-22 production by CD4+ cells is mainly mediated by cytokines like IL-23, IL-21, IL-12, IL-1β, IL-7, IL-6, TNF-α (Tumor Necrosis Factor) and some other molecules like Notch, RORyt and aryl hydrocarbon receptor ligand like FICZ [6-formylindolo[3,2-b] carbazole (31, 32)]. In Th17 cells, TGFβ (transforming growth factor beta), in the presence or absence of IL-6, plays an important role in the production of both IL-17 as well as IL-22 (9). Furthermore, in peripheral blood, another major source of IL-22 production is the Th22 cells. In the presence of TNF-α and IL-6, naïve T cells can be differentiated into Th22 cells and produce IL-22 (33, 34), which play an important role in tissue remodeling (35, 36). Notch receptor family and AhR androgen ligands mediate Th22 cells to produce IL-22 (37, 38). Transcription factor HIF-1a has also been reported to stimulate CD4+ cells to produce IL-22 (39). Furthermore, IL-22 Is also secreted by natural killer T cells, γδT cells, and CD8+ T cells when they are activated, particularly when IL-23 is present (40). Innate lymphoid cells are another key producer of IL-22 which include LTi cells (lymphoid tissue inducer cells), NCR (natural cytotoxicity triggering receptor) positive cells and NK cells (41–43). Non-hematopoietic cells, macrophages, and monocytes do not produce IL-22 in humans (28).

Although ubiquitous expression of IL-10R2 is found throughout the body, the defining factor for the cellular sensitivity of IL-22 is expression of IL-22R1 (44). It is now well-recognized that T cells, B cells, Natural Killer cells, and other immune cells are not targeted by IL-22 (28). Instead, IL-22R1 expression is seen in tissue cell types that primarily construct epithelial barriers like bronchial epithelial cells (45), intestinal epithelial and subepithelial myofibroblast (46), skin keratinocytes and fibroblast (47), and thymic epithelial cells (48). Other than that IL-22 receptors are also expressed in hepatocytes (10), pancreatic acinar cells (49) and islet β-cells (50), kidney epithelial cells (51), and specific tissue resident stem cells (52). Varied levels of IL-22 has been observed depending upon the tissues and extracellular environment. A significantly reduced expression of IL-22 has been noticed in intestinal mucosa of ulcerative colitis patients due to increased concentration of TGF-β as compared to healthy colon tissues (53). On the other hand, over expression of Hes1 enhanced the Il-22 transcription in Intestinal Epithelial Tissue via STAT3 phosphorylation (54).

## IL-22 Biological Effects

**Figure 2:**
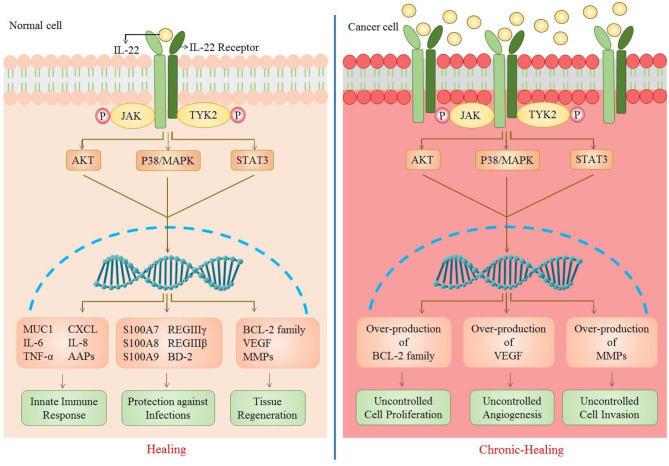
Role of IL-22 in normal vs. cancer cell. As IL-22 attaches to its receptor, a number of downstream signaling pathways are activated that further result in the upregulation of many genes. This causes an enhancement of biological functions including innate immune response (MUC1, IL-6, TNF-a, CXCL, IL-8, AAPs), protections against infections (S100A7, S100A8, S100A9, REGIIIy, REGIIIb, BD-2), and tissue regeneration (Bcl-2 family, VEGF, MMPs). The up-regulation of these genes is caused by multiple signaling pathways (JAK/STAT3, P38/MAPK, AKT). However, in the case of increased IL-22 or IL-22R, there is persistent activation of such signaling pathways which results in over-production of cell survival, angiogenic and metastatic genes [CXCL, chemokine (C-X-C motif) ligand 1; MUC1, Mucin1; AAP, Amyloid Precursor Protein; S100A7/8/9, S100 calcium-binding protein A7/8/9; REG, regenerating islet-derived protein 3; BD-2, beta-defensin 2; VEGF, vascular endothelial growth factor; MMPs, matrix metalloproteinases].

IL-22 carries out a number of biological effects including innate immune response, protective role against pathogens, and tissue regeneration. However, over generation of such responses can gradually lead to carcinogenesis (Figure 2).

### Innate Immune Response

Like other cytokines, IL-22 plays pivotal role in generating innate immune response against various infections. These responses involve in maintaining homeostasis at epithelial surface, recruitment of other immune cells by provoking inflammation and mucus production against pathogens (Figure 2).

#### Inflammation

IL-22 exhibits many pro-inflammatory effects in order to generate an immune response. In Colo205, a colon epithelial cell line, IL-22 effectively generates an acute phase response by up-regulating expression of serum amyloid protein, α-chymotrypsin, and haptoglobin (55). In response to any tissue damage or invasion, IL-22 is known to recruit neutrophils toward the site of infection as an inflammatory response. IL-22 attained it by persuading the production of certain chemokines like CXCL1, CXCL2, and CXCL5 in primary human keratinocytes (56). It has been observed both in mice and humans that in order to orchestrate inflammatory response, IL-22 affects the production of other pro-inflammatory cytokines which include IL-6, IL-8, and TNF-α. Such cascade reactions have been reported in hepatic stellate cells, myofibroblasts, and keratinocytes (57, 58). In cell line like human keratinocyte cell line HaCaT, IL-22 enhances NLRP3 inflammasome which yields IL-1β (59). This further insinuates IL-22 provoked oxidative signaling, achieved by Nicotinamide Adenine Dinucleotide Phosphate (NADPH) oxidase mediated STAT3 activation. This might synergize with IFN-γ to generate nitric oxide species (60). Pro-inflammatory activity performed by IL-22 has also been associated with several inflammatory diseases such as psoriasis and atopic dermatitis (61, 62). Furthermore, IL-22 mediated airway inflammation promotes atopic march which usually proceeds to asthma (63). However, the modulation of IL-22 expression done by IL-22BP lessens the damaging effect of inflammation in murine model (64).

On the other hand, in conditions like hepatitis, asthma, inflammatory bowel disease and cholangitis, anti-inflammatory response of IL-22, to counteract the disparaging effect of immune response, has also been observed (65–68).

Deficiency of IL-22, as observed in patients and Reconstituted Human Epidermis (RHE) tissues, leading to chronic inflammatory acne inversa points toward its anti-inflammatory role in homeostatic condition (69). It allows us to conclude that IL-22 is capable to perform both pro- and anti-inflammatory responses, depending upon tissue microenvironment including cytokine milieu (70).

#### Mucus Production

Mucus layer in certain epithelial cells serves as innate mechanism against both bacterial as well as viral infections and also for other toxins released by parasites (71). IL-22 serves to up-regulate a number of mucus associated genes, such as MUC1, MUC3, MUC10, and MUC13 in colonic and tracheal epithelial cells (46, 72). IL-22 along with another cytokine IL-17A upholds production of pulmonary mucus as a control against Gram negative pathogens like *Klebsiella pneumonia* (72). STAT3 activation via IL-22 gene delivery within colonic epithelial cells restitutes mucus-producing goblet cells. It rapidly ameliorates local intestinal inflammation in acute colitis murine model (46). Decline in IL-22 production is known to play role in HIV associated immunopathogenesis (73). It is suggested that IL-22 may persuade resistance against HIV infection in people who are exposed to virus several times by enhancing mucus production (74). As IL-22 enhances and maintains mucosal barrier integrity, its transient inhibition is also suggested for mucosal vaccines to increase T cell response (75).

### Protective Role Against Infections

The well-documented role of IL-22 is prevention and defense against bacterial and other parasitic infections in various organs. It also protects against viral infections by reducing the follow-up infections and assisting in tissue retrieval (Figure 2).

#### Bacterial Infections

Production of antibacterial proteins is one of the main steps of IL-22 mediated immune responses in murine model (76). Commonly IL-22 elevates antimicrobial defense by up-regulating expression of certain antibacterial genes like psoriasin (S100A7), calgranulin-A (S100A8), calgranulin-B (S100A9), and Beta-defensin 2 (BD-2) independently or in combination with IL-17A of IL-17F as per studied in primary human keratinocytes as well as in patients' skin biopsies and blood plasma (29, 77). However, various studies on murine model demonstrated that IL-22 induced STAT3 controls bacterial growth in intestinal epithelial cells by enhancing expression of proteins specifically RegIIIγ (regenerating islet-derived protein 3) and RegIIIβ (78). When present at mucosal surfaces, RegIII proteins serve to exhibit antimicrobial action against gram-positive bacteria by binding to the peptidoglycan moieties of bacteria and induce damage to the bacterial cell wall and separate microbiota from intestinal epithelial cells to maintain a symbiotic host-bacterial relationship (79, 80). Similarly, recombinant porcine IL-22 activates STAT3 signaling to protect against *Escherichia coli* infection (81).

IL-22 plays crucial role in protecting the skin, gastrointestinal and respiratory tract from both pathogenic and commensal bacterial infections. Its activity is critical for regulation of gut microbiota. Depletion of IL-22 producing innate lymphoid cells causes peripheral diffusion of intestinal commensal bacteria *Alcaligenes*. This dissemination is sufficient to induce systemic inflammation which has potential to facilitate Crohn's disease and progressive hepatitis C viral infection (82). Similarly, upon respiratory infection with *Streptococcus pneumonia*, type 3 lymphoid cells (ILC3) accumulate in lung tissue. In murine model, this amassing of ILC3 is followed by production of IL-22 that defends against lethal infection (83). Knocking out of IL-22 binding protein (IL-22BP), which reduces IL-22 activity, is found to decrease pneumococcal murine lung infection (84). Natural killer cells are reported to provide immunity against *Mycobacterium tuberculosis* (Mtb) by releasing IL-22 in infected patients, which constrains intracellular bacterial growth by accelerating phagolysosomal activity (85). IL-22 also provides immunity against rapidly emerging Mtb HN878 strain in mice (86). Mechanism underlying this inhibition involves IL-22 dependent up-regulation of calgranulin A, an intracellular signaling molecule that induces phagolysosomal fusion (87). IL-23 dependent IL-22 production is shown to protect against *Salmonella enterica*, which is known to cause diseases ranging from minor gastroenteritis to serious systemic infections. In absence of IL-22, murine model developed liver necrosis upon *Salmonella* infection (88). In case of chronic *Salmonella* gastroenteritis model, antibody mediated IL-22 neutralization disrupted the epithelial barrier of intestine and increased the production of pro-inflammatory cytokines in porcine intestinal epithelial cells (81). IL-22 mediated anti-bacterial activity against *Salmonella* is carried out by phagolysosomal fusion in intestinal epithelial cells (89). That's why prompt up-regulation of IL-22 by dendritic cells is seen at site of *Salmonella* infection to cause resistance against salmonellosis (90). Furthermore, relative deficiency of IL-22 is seen in Acne Inversa patients which occurs due to chronic inflammation caused by persistence cutaneous bacterial infection (69).

However, contrary to IL-22 protective role against bacterial infections, one study done on BALB/c mice, implied its dispensable role in immunity against opportunistic pathogens like *Mycobacterium avium* and *Mycobacterium tuberculosis* (91). Interestingly, IL-22 up-regulation after *Listeria monocytogenes* infection was seen in murine model, though, no clear effects on both primary and secondary bacterial infection were observed (92). Its pro-inflammatory activity is also connected with bacterial spread leading to organ failure in septic peritonitis observed in murine models (93). Elevated level of IL-22 in plasma has also been related with psoriasis, which markedly reduced after anti-psoriatic therapy. In this case, IL-22 mediated over-expressions of antibacterial proteins become the cause of disease severity (77).

Ample data elucidate how IL-22 exhibits antibacterial activity by inducing various innate defense mechanisms in epithelia. However, in some cases its production is inferred to play roles other than clearance of bacterial infection, which may also lead to pathogenicity.

#### Fungal Infections

IL-22 contribution to protect against fungal infection was first suggested by a study conducted on mice. *Aspergillus fumigatus* infection in lungs was reported to be limited by IL-22 mediated immunity (94). Acute *A. fumigatus* exposure instigates IL-7 and IL-21 which further regulate the IL-22 production to carry out anti-fungal effects (95). IL-22 along with IL-17F is a vital natural defender against Chronic Mucocutaneous Candidiasis (CMC). IL-22 provides first line of defense against *Candida albicans* as observed in humans and murine models, which comprises monitoring of fungal growth. And it is required to seize dissemination of intragastric infection of *C. albicans* to other organs like stomach and kidney (96, 97). In addition, IL-22 exhibit mild role in protecting against cutaneous candidiasis in mice (98). Likewise, mice deficient in IL-22 were slightly susceptible to oropharyngeal candidiasis, indicating a role of IL-22 against oral candidiasis (99). Contrary to given data, IL-7 mediated IL-22 production orchestrates immunopathogenic reactions in fungal asthmatic patients (100, 101).

#### Viral Infections

According to previous studies, IL-22 was known to be incapable of having direct antiviral response, though its profound role in post-viral infection tissue repair is well-described. However, recently IL-22 is identified to exhibit a novel role in regulating antiviral T cell response in non-lymphoid and lymphoid organs in the course of acute and persistent viral infections (102). Upon influenza viral infection, Dendritic Cells (DCs), and activated Natural Killer T Cells (NKT cells) are ultimate source of IL-22 (103). This rapid production of IL-22, in murine model is implied to retain beneficial role in preserving bronchoalveolar, tracheal, and lung epithelial integrity (104), and post-infection tissue restoration (105). IL-22 production in sublethal H3N2 influenza virus infection is known to limit lung inflammation and specifically subsequent secondary bacterial infections (106, 107). Human immunodeficiency virus (HIV) and other similar viruses like Simian immunodeficiency virus (SIV) spread via multifocal injury of gastrointestinal epithelial barrier. Perturbance in mucosal immunity on onset of SIV infection is known to be correlated with loss of IL-17 and IL-22 expression (108). Similarly, impairment in IL-22 production due to Th22 depletion in mucosal gut is a significant factor in HIV mediated mucosal immunopathogenesis (73). Anti-HIV candidate Abx464 reduces intestinal inflammation by stimulating activated macrophages to produce more IL-22 in mice model (109). Inoculation of IL-22 along with IL-18 to murine model reiterated the capability of flagellin to inhibit or eradicate Rotavirus (RV) infection (110). In addition, IL-22 and Interferon lambda (IFN- λ) both retain the integrity of intestinal epithelial cells to curtail RV replication (111). Kaposi's sarcoma-associated herpesvirus (KSHV) latency depends upon viral mediated IL-22 down-regulation to suppress antiviral response (112). However, in another study performed on mice pathogenic role of IL-22 is explicated where IL-22 signaling is reported to exacerbate lethal West Nile Virus (WNV) encephalitis prospectively due to WNV neuroinvasion (113). Correspondingly, IL-22 deficient mice upon Zika Virus inoculation revealed lessened weight loss, systemic inflammation, neurological disorders and mortality (114).

IL-22 up-regulation is reported multiple times in liver patients with chronic Hepatitis B Virus (HBV) or Hepatitis C Virus (HCV) infection (115–117). IL-22 can manifest a protective as well as pathogenic role in the liver, by impediment of apoptosis and stimulation of stem cells. Both outcomes can employ their protective tasks by increasing number of hepatocytes or can exhibit their pathological effects by enhancing production of chemokines, matrix metalloproteinases (MMPs), and neutrophil recruitment toward the liver in humans and mice (118, 119). IL-22 pro-inflammatory response following HBV recognition by liver T cells might play beneficial role in infection (120). IL-22, mainly manufactured by hepatic γδ T cells is shown to attenuate liver injury in case of adenovirus-infected murine model (121). In contrast with IL-22 beneficiary effect, its association with prognosis in chronic liver inflammation, fibrosis and Hepatitis B virus-related acute-on-chronic liver failure (HBV-ACLF) have also been studied (122–124). HCV induced IL-22 up-regulation in liver cells does not itself regulate antiviral proteins and HCV replication in cell lines (125). Though, protection against liver fibrosis and cirrhosis caused due to HCV infection is seen which can be varied due to genetic alteration in IL-22 gene (4, 126).

### Wound Healing and Tissue Regeneration

IL-22 binding with its receptor complex stimulates downstream signaling pathways that activate cell survival genes. Up-regulation of cell proliferation genes supports tissue redevelopment and wound healing [Figure 2; (127)]. IL-22 mediated myofibroblast differentiation and production carry out skin wound healing process in mice (128). Upon skin inflammation, increase in IL-22 expression aids keratinocyte proliferation and migration toward site of injury. Meanwhile, during the process of wound healing, keratinocyte differentiation is repressed by IL-22 (127, 129, 130). Deficiency of IL-22 in injury has shown compromised granulation, ECM production and formation of tissue and wound contraction. Primary dermal fibroblast bears IL-22R1 which, when bind with IL-22, stimulates downstream JAK/STAT pathway. Following dimerization and translocation of STAT molecules into nucleus, results in enhanced production of ECM, fibronectin, and collagen. Therefore, in IL-22 deficient murine model, faulty wound contraction, and ECM production are observed (131). Likewise, in another study, absence of IL-22 resulted in defective recovery from DSS-caused intestinal injury in mice. Alternatively, a gene delivery system increasing the IL-22 expression in intestinal mucosa enhanced intestinal repair (78). A separate study elucidates that IL-22 deficiency can also alter the intestinal microbiota thus worsening disease severity. This study also signifies the importance of IL-22 in maintaining the balance between microbiota of the intestine and intestinal immunity (132).

IL-22 is also recognized to play key role in tissue regeneration after an injury. Hepatocyte proliferation serves as a requisite process in order to undergo tissue regeneration following hepatectomy. In that scenario, post-surgery IL-22 activation helps in orchestrating cell survival and proliferation genes (133, 134). Accumulating evidence through studies on mice suggest that IL-22 mediated liver regeneration is carried out in collaboration with IL-6 and TGF-α signaling cascades (135). Similarly, IL-22 induced STAT3 triggering activates intestinal stem cells to regenerate epithelium (136, 137). Up-regulated IL-22 expression during thymic recovery allows for the renewal and regeneration of thymus (48). In case of allogeneic hematopoietic cell transplant, donor T-cell derived IL-22 not only regenerates insulted thymus, but also reduces chronic graft-versus-host disease (GVHD) likelihood. It is achieved by IL-22 directed enhanced expression of Aire, an autoimmune regulator in murine model (138). Upon influenza virus infection, tracheal epithelial cells underwent proliferation and regeneration in response to IL-22 expressed in wild-type mice (104). Though a regenerative potential of IL-22 in renal tubular injury is well-defined, however, in glomerular disease, redundancy in IL-22 expression was observed (139). In addition, IL-22 has been shown to stimulate β-cells expression of Reg proteins in pancreas. Reg proteins are believed to participate in regenerating islets (140). However, regenerative cell survival signaling of IL-22 has a profound potential to shift toward tumor formation, when over-activated in uncontrolled manner. It was also observed that IL-22R deficient mice had a delay in wound healing. IL-22, along with IL-20 and IL-24 enhances wound healing in type II db/db diabetic mice by inducing genes involved in re-epithelialization, tissue remodeling and innate host defense mechanisms from injured skin (141).

Cellular sources of IL-22 has been observed to vary in different tissues. As per studies, Th-22 cells represent the major source of IL-22 in skin (34). Whereas, NK cells contribute as releasing factor of IL-22 in gut cells at time of inflammation in patients like ankylosing spondylitis (142). Moreover, STAT-3 mediated IL-22 signaling is associated with mucosal wound healing in epithelial cells of intestine (78). Similarly, Lymphoid tissue inducer (LTi) is known to be a source of IL-22 in fetal lymph nodes and spleen for its regenerative functions (143). Higher levels of IL-22 in keloid scars have also been observed, however, its exact role whether as healing or pathological factor is still needed to be explored (144).

## IL-22 in Cancers

Despite the well-documented involvement of IL-22 in inflammatory responses leading to wound healing and tissue regeneration, in certain circumstances, where there is a disturbance in the intricate balance between repair and damage, IL-22 can serve in the direction of carcinogenesis. This is due to the participation of pathways favoring cellular proliferation and survival that are common in both wound healing and carcinogenesis. Consequently, IL-22 has the potential of promoting carcinogenesis in organs that are responsive to IL-22 due to the presence of its receptor (Figure 2).

### Colorectal Cancer

**Figure 3:**
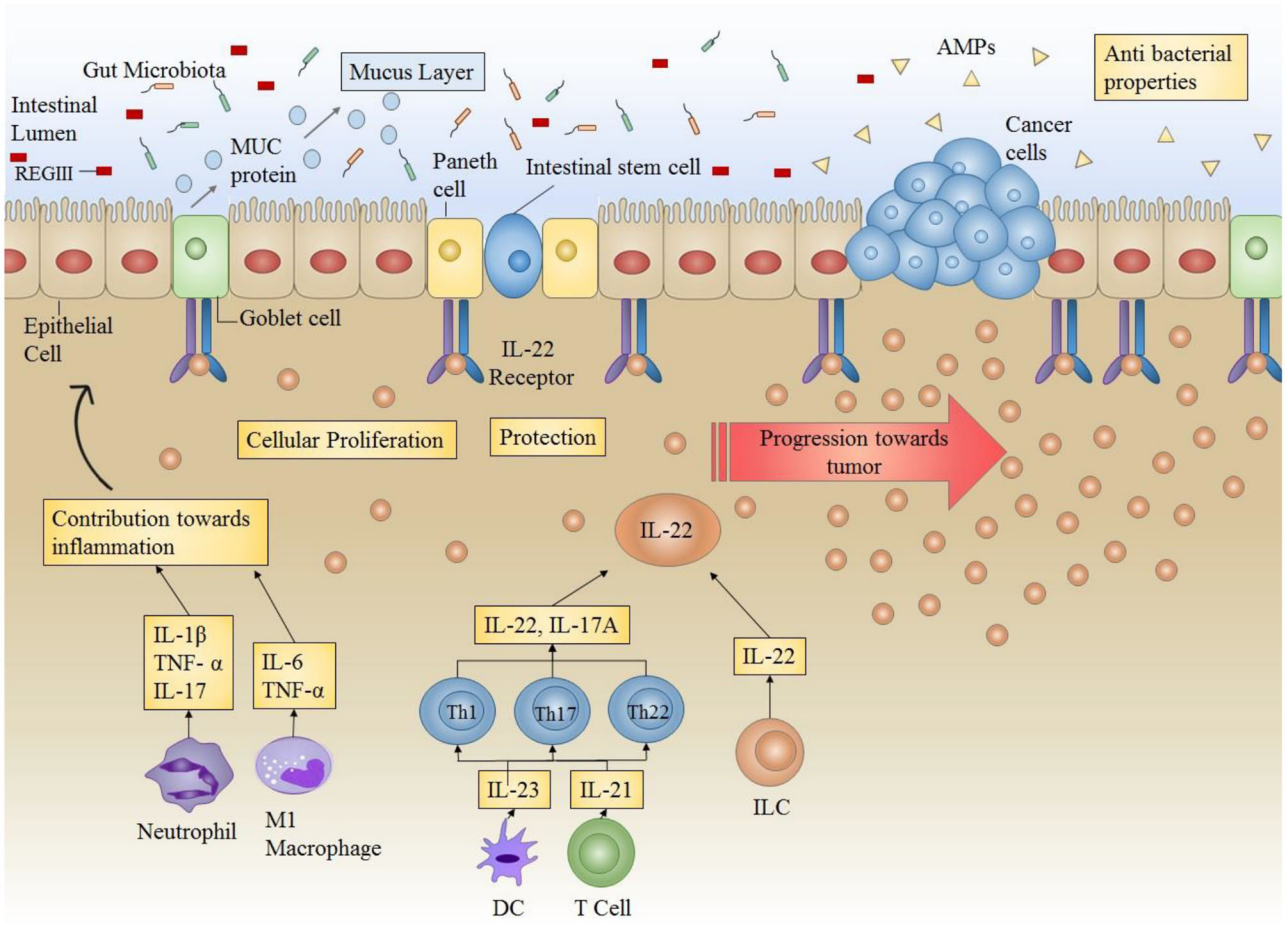
Role of IL-22 in the intestinal microenvironment: Under normal circumstances, IL-22 plays a very important role in providing protection against infections by increasing the production of AMPs (antimicrobial peptides) (including REGIII) thus enhancing antibacterial competence. IL-22 also increases mucus secretion by producing mucins (MUC) in order to ensure protection of epithelial layer and maintenance of epithelial barrier. Furthermore, IL-22 also directly acts on epithelial stem cells to enhance cellular proliferation. IL-22 in conjunction with other cytokines maintains the overall environment for protection against infections and inflammations. However, in case of prolonged or uncontrollable expression (as in colitis), IL-22 can result in progression of tumor formation.

Chronic intestinal inflammation and prolonged tissue damage are two of the main factors that predispose the colon mucosa to carcinogenesis. This can be attributed to the underlying mechanisms involved in wound healing and their potential to lead toward carcinogenesis in case of a disrupted balance. This can be based on the fact that signaling pathways driving the processes of tissue repair and cancer development share many similarities and several common factors. Therefore, in order to evade the development of cancer, tight control should be in place to balance the factors mediating tissue repair in case of tissue damage. A long term tissue healing response is required in the presence of persistent tissue damage or chronic inflammation. However, it can ultimately result in fibrosis and carcinogenesis. Therefore, development of cancer can be deemed as an outcome of excess and abnormal healing occurring due to failed regulation of signaling (Figure 3).

Increasing evidence shows an involvement of IL-22 in progression of colorectal carcinogenesis. Key cellular sources responsible for producing IL-22 in the setting of colon cancer include ILCs and Th22 cells. IL-22 producing CD4+ T cells have been shown to enhance the stemness of colorectal cancer (145). Colonic dendritic cells, irrespective of their maturation level are responsible for secreting high IL-22 levels (146). In murine models, boosted levels of IL-22 reveal an indispensable role of this cytokine in the enhancement of tumor burden and decline in survival rate (147). Furthermore, by augmenting cellular proliferation and up-regulating inflammation, ILCs have proven to be essential in transitioning intestinal inflammation into colorectal cancer (148, 149).

A direct association has been reported between stagings of colorectal cancer with IL-22. IL-22 administration results in the acquisition of chemoresistance in human colorectal cancer cell lines (150). In a murine model of colitis mediated carcinoma, deficit in IL-22 ensured the development of fewer tumors. At the molecular level, IL-22 promotes carcinogenesis of colon cancer cells by activating STAT3, AKT, MAPK, and NFκβ. The enhancement of tumor cell proliferation also occurs as a result of modification of p16 and p21 promoters which are cell cycle checkpoint genes, thus resulting in heightened carcinogenicity (151, 152). IL-22 demonstrates a dual nature in facilitating carcinogenesis. The reduced expression of this cytokine outcomes in hampered tissue repair thus prolonging the process of inflammation and ultimately results in carcinogenesis. On the other hand, enhanced IL-22 expression can extend tissue regeneration process and also stimulate the development of colon cancer (25). In conformity with studies, the removal of ILCs in murine model of inflammation mediated carcinogenesis of colon cells shows a decrease in tumor formation. Similarly, anti-IL-22 antibody administration restored the symptoms associated with colitis along with the reduction of cancer burden (52). Based on these findings, it is concluded that a tight regulation and controlled release of IL-22 is required for effective wound healing that does not progress to carcinogenesis.

### Gastric Cancer

IL-22 has been revealed to act as a pro-tumor cytokine in the cancers associated with gastrointestinal tract. The infiltration of cellular sources of IL-22 including Th22 and CD4+ T cells in the intratumoral tissue increases, corresponding to the stage of tumor (153–155). Patients with gastric tumors have also been shown to have increased levels of circulating T cells secreting IL-22 as well as IL-17, thus showing a positive association with tumor progression (154). In gastric cancer patients, IL-22R1 expression is augmented that indicates a positive correspondence with the stage of cancer and lymphatic invasion (156). Chromosomal mutations including single nucleotide polymorphisms and chromosomal gains have also been reported in gastric cancers. A SNP was found in the IL22 locus which was linked to an increase of about 2.5-folds in the risk of development of gastric tumors (157). Chromosomal gains at the IL22RA1 locus 1p36.11 are also observed which suggests that gastric cancer cells show increased responsiveness to IL-22 due to a high copy number of IL-22R1 (158). IL-22 gene polymorphisms were found to increase the susceptibility of developing gastric cancer in a study that was conducted on Asian population (159). Furthermore, IL-22 has also been described to aid metastasis of gastric tumor cells via increasing the expression of matrix metallopeptidases. Elevated levels of matrix metallopeptidases enhance the invasiveness of tumors (160). Various studies have shown boosted levels of IL-22 in chemotherapy resistance patients to FOLFOX4 adjuvant chemotherapy. Moreover, IL-22 mediated drug resistance has also been observed in 5-Fluorouracil and Oxaliplatin treated colon cancer cell lines (150).

### Pancreatic Cancer

**Figure 4:**
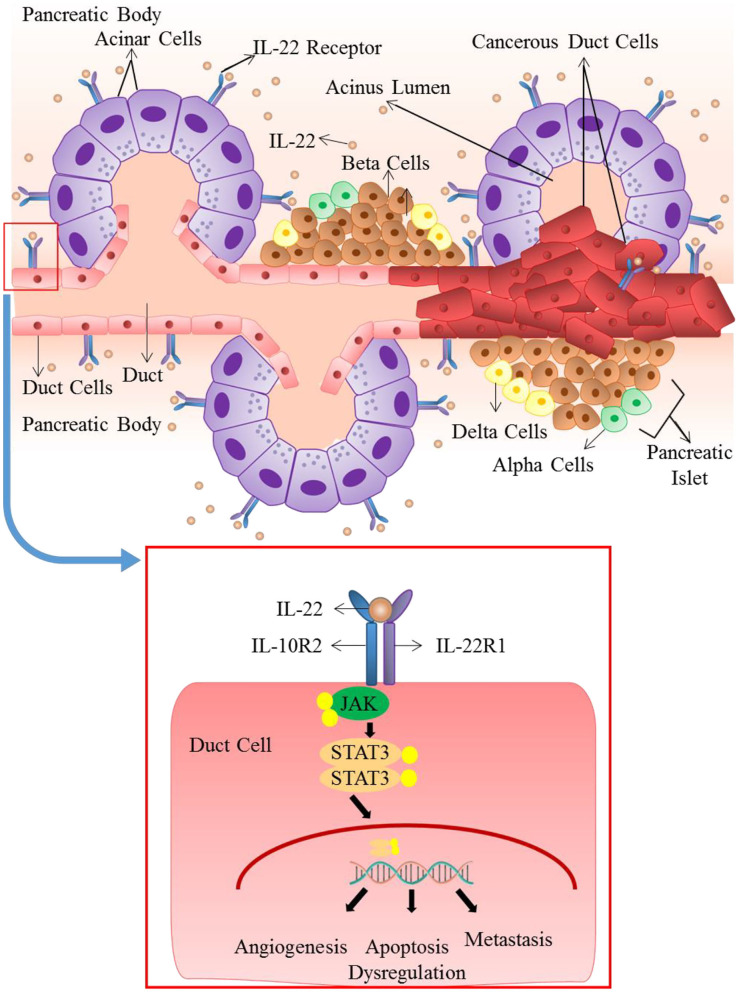
Role of IL-22 in pancreatic ductal adenocarcinoma: IL-22 mediated ductal adenocarcinomas of the pancreas expresses elevated levels of IL-22 and IL-22R1. Dysregulated and persistently elevated levels of IL-22 stimulates the production and release of various tumor enhancing factors through activation of JAK/STAT3 pathway. It results in apoptosis dysregulation, angiogenesis and metastasis that lead to advancement of cancer.

In humans, the highest expression of IL-22R occurs in the pancreatic acinar cells, therefore pancreatic cells are one of the main targets of IL-22 (49, 50). Many studies reveal that in pancreatic inflammation, IL-22 elicits a protective role (161, 162). This is achieved through up-regulation of anti-apoptotic genes including RegIII, thus ensuring the survival of cells and stimulating tissue repair. Mice deficient in IL-22 exhibit a worsening of tissue damage in case of pancreatitis. And this results in an increase in fibrosis (77). However, in the instance of persistently increased IL-22 expression and signaling dysregulation, this protective role can be easily manipulated into a carcinogenic one (163). In pancreatic cancer cell lines, production and release of various tumor enhancing factors including VEGFα, Interleukin 10 and TGF-β are stimulated by IL-22. IL-22 mediated ductal adenocarcinomas of the pancreas expresses elevated level of IL-22 and IL-22R1, concomitant with enhanced MMP production and invasion of lymph nodes. This illustrates the pro-tumor functions of IL-22 [Figure 4; (164, 165)]. Elated expression of systemic IL-22 positively correlates to poor prognosis of patients undergoing resection for pancreatic ductal adenocarcinomas. Therefore, the increased intra-tumoral infiltration of cellular sources of IL-22 including CD4+ cells and Th22 cells elevated IL-22 levels entail a poor patient survival outcome (119). IL-22 has been shown to increase the tumorigenicity and stemness of pancreatic cancer cells, through JAK/STAT3 signaling (166). IL-22 increases the expression of VEGF, anti-apoptotic gene Bcl-X and some immunosuppressive cytokines (167). Likewise, through STAT-3 dependent up-regulation of MMP9, *in vitro* IL-22 has been shown to enhance the metastatic potential of pancreatic cancer cell lines (168).

### Skin, Lung, and Brain Cancer

In spite of the fact that IL-22 has a significant function in autoimmunity and the inflammatory responses of skin, involvement of IL-22 in skin cancer is rarely documented. One study reports, an increased infiltration of cells secreting IL-22 specifically Th22 cells in squamous cells carcinoma of skin. This infiltration and recruiting of Th22 cells to the site of cancer was partially due to MMP10 and S100A15 (169). A higher number of these cells were also found in basal cell carcinoma. IL-22 increased the proliferative as well as migratory potential of both basal cell carcinoma as well as squamous cell carcinoma of skin through STAT3 pathway and AKT (170). CSA (Cyclosporin) is involved in increasing the Squamous Cell Carcinoma (SCC) by favoring polarization of Th22 which results in increased IL-22 production and thus the blockage of IL22 by using anti-IL-22 antibody could potentially become a viable therapeutic option (31). Keratinocyte's response to IL22 is increased by UVB resulting in skin inflammation (171). Another *in vitro* study reveals that IL-22 and IL22R1 expression is enhanced in tumorous and peri-tumorous tissues, where cellular proliferation may be encouraged by IL-22 (172). It has also been reported that IL-22 elevated in lung cancer and renders lung cancer cells resistant to chemotherapy (173). In patients with malignant pleural effusions in non-small lung cancer, increased IL-22 levels have been associated with a poorer prognosis and survival rate (173). IL-22 has also been shown to be increased in non-functioning and prolactin-secreting macroadenomas of the pituitary (174). Murine model of glioma illustrates that increased IL-22 levels in brain can worsen the symptoms. This may occur due to enhanced IL-6, TNF, and IL-1β production stimulated by IL-22. Correspondingly, by using anti-IL-22 antibody or in IL-22 knock-out mice, the decreased IL-22 expression exhibited protective roles in the development of glioma (175).

### Liver Cancer

IL-22 has an imperative role in liver regeneration and liver tissue healing. IL-22 shows the ability to reverse the damages caused to liver by activating wide range of healing agents and signaling pathways (10, 67, 135, 176–179). However, in the setting of chronic inflammation as in the case of chronic HCV and HBV infections, persistently elevated levels of IL-22 seem to aid the process of carcinogenesis (180). Hepatocellular carcinoma (HCC) tumor-infiltrating lymphocytes (TILs) are reported to be enriched with IL-22 in consort with elevated expression of IL-23 and IL-22BP in cancerous tissue. Enhanced level of IL-22 has been positively correlated with formation and staging of tumors (181). Reportedly, IL-22 stimulates STAT3 phosphorylation which elevates the expression of proto-oncogenes in HCC (168). A study showed that IL-22 liver specific transgenic murine model was more prone to develop diethylnitrosamine-induced tumorigenesis (124). In agreement to this, when IL-22 expressing TILs derived from HCC patients were introduced into lung cancer xenograft murine model, it orchestrated oncogenic signaling pathways in the tissue (181). Overall, IL-22 elevated levels in murine models and liver cancer patients point toward its vital role in liver tumor development and progression.

## Manipulation of IL-22 Axis for Therapeutic Purposes

The IL-22—IL-22 subunit (IL-22R1) axis has shown a high potential clinical relevance in inflammatory diseases like psoriasis, ulcerative colitis, liver and pancreatic damage, graft-versus-host disease, certain infections, and tumors.

A direct IL-22-neutralizing antibody, ILV-094, has completed Phase I and II trials for psoriasis and rheumatoid arthritis, respectively. Neutralization of IL-22 may improve disease control and quality of life for late stage disease patients by reducing metastasis, chemoresistance and inflammation associated with cancer (182). Single i.v. dose of recombinant IL-22 (3.5 μg per mouse) resulted in Attenuation of acetaminophen (paracetamol)-induced liver damage (183). In liver, *in vivo* IL-22 cDNA delivery resulted in protection from ConA-, carbon tetrachloride-, and Fas-induced liver damage (184). These approaches show great potential for future therapeutics aimed at enhancing tissue repair or preventing cancer development.

## Conclusion and Future Directions

T helper cells (including Th1, Th17, and Th22) are the primary producers of IL-22. Innate lymphoid cells are another key producer. IL-22 is an inflammatory cytokine so it is produced as a result of tissue injury and is clearly detectable in plasma following local tissue inflammation. Therefore, in inflammatory conditions, like in tissue injury, or infection, IL-22 level is significantly enhanced. Environmental factors, including cytokines (mentioned in section IL-22 Sources and Targets as the causes for induction of IL-22 production), metabolites, and oxygen play an important role in regulating the time and place of increasing IL-22 expression as well as shutting it down (77). In the case of acute inflammation, IL-22 generally plays a protective role like in skin, liver, colon, pancreas, lungs, and epithelial cells, however, in the case of chronic inflammation, this protective role can be converted into a pathological one. The epithelial cells of many organs, liver cells, fibroblasts, and pancreatic cells are considered to the principal target of interleukin-22 (77). IL-22/IL-22R1 axis alter signaling in target cells. The epithelial cells respond by enhancing the expression levels of many different proteins including anti-bacterial proteins like BD-2, S100A7-9, LCN2 (lipocalin-2) chemokines that attract granulocytes (CXCL 1, 5, and 8) and some MMPs. In the respiratory cells of colon and lungs, IL-22 causes the target cells to generate MUC-1. In skin epithelium, IL-22 has shown to decrease the expression of certain proteins implicated in cellular differentiation of epithelial cells (for example keratin 1 and keratin 10, desmocollin 1, and involucrin). The chief targets of IL-22 are the pancreatic cells and liver cells, where IL-22 upregulates Bcl-2, Mcl-1 (important antiapoptotic proteins), mitogenic proteins (like p21 and RBL2), and Bcl-XL. Anti-bacterial proteins like REG3 are also upregulated (178). Additionally, in liver cells, IL-22 stimulation also enhances the levels of some acute phase proteins, including haptoglobin LPS binding protein and serum amyloid A (135, 185). In rheumatoid arthritis patients, CC chemokine ligand 2 (a chemokine that attracts monocytes), and RANKL (receptor activator of NF-kB ligand), that stimulates monocytes to be differentiated into osteoclasts, are also increased by IL-22 (186). No effect of IL-22 on activated or resting immune cells is considered to be an important property of IL-22. IL-22 also synergizes with other cytokines like IL-17 and interferon gamma to elicit its role Table 1.

**Table 1:** Table demonstrates how the role of IL-22 can differ in the setting of acute vs. chronic inflammation and how it can convert the protective role of IL-22 into a pathological one.

**Inflammatory condition**	**Role**	**Tissue/Organ**	**Effect**	**References**
Acute inflammation	Protective	Liver, progenitor cells Pancreas Lung Epithelial Cells Skin Colon	Proliferation	(104, 105, 127, 133, 134, 136, 137, 161, 162, 185)
		Skin Intestinal mucosa cells	Wound healing	(78, 127, 128, 131, 132)
		Liver Kidney Lung Thymus Pancreas	Regeneration	(48, 104, 133, 134, 139, 161, 162)
		Keratinocytes	Cell motility	(127, 129, 130)
		Skin keratinocytes Intestinal epithelial cells Lung	Antimicrobial	(29, 77, 78, 83, 89)
		Hepatic stellate cells Keratinocytes Colon Myofibroblasts Epithelial cells	Pro-inflammatory	(127, 128, 136, 137, 190)
		Intestinal epithelial cells Goblet cells	Mucus production	(71, 72)
Chronic inflammation	Pathological	Colon	Uncontrolled proliferation Tumor growth	(145–152)
		Lung	Tumor growth	(173)
		Gut	Potential risk for tumor Tumor growth	(153–160)
		Liver	Tumor growth	(124, 168, 180, 181)
		Pancreas	Tumor growth	(163–168)
		Skin	Tumor growth	(31, 169–171)
		Brain	Tumor growth	(174, 175)

*In acute inflammatory responses, IL-22 is important for proliferation, wound healing, and regeneration. While the role of IL-22 can become pathological and result in carcinogenesis in the case of chronic inflammation*.

However, in some cases, IL-22 signaling differs from reparative conditions and causes pathological conditions like inflammatory diseases and cancers due to dysregulation or increased levels like in chronic inflammation. All of these above mentioned pathways, that are essential for wound healing, regeneration and protection, become the cause of pathological conditions when persistently expressed (as in chronic inflammatory conditions). Furthermore, the levels of IL-22/IL-22R1 axis also differ in early and late cancers which shows its potential to be used as a staging marker in the future. The influence of IL-22 on hyperplasia, adenoma, early carcinoma, and late carcinoma stages of cancer was investigated using IL-22^−/−^/MMTV-PyMT spontaneous breast cancer mouse model showing that it is necessary for malignant transformation of cancer cells which is the critical stage for metastasis. Inhibition of IL-22 can hinder cancer cell malignancy (187). Similarly the infiltration of cellular sources of IL-22 including Th22 and CD4+ T cells in the intratumoral tissue increases, corresponding to the stage of tumor (153, 155). Patients with gastric tumors have also been shown to have increased levels of circulating T cells secreting IL-22 as well as IL-17, thus showing a positive association with tumor progression (154). In gastric cancer patients, IL-22R1 expression is augmented that indicates a positive correspondence with the stage of cancer and lymphatic invasion (156). Furthermore, IL-22+ immune cell frequency was also higher in HBV-infected patients with liver cirrhosis than in non-cirrhotic patients in positive correlation with cirrhotic stage score (123). In colon cancer, enhanced IL-22 expression is associated with poor prognosis and an enhanced tumorigenesis, with protective role shown by IL-22 binding protein (188). IL-22 and HOXB-AS5 (a long non-coding RNA located in HOX gene clusters) were upregulated in the serum and tissues of Breast Cancer patients and were associated with clinical stages which showed that the IL-22-HOXB-AS5-PI3K/AKT functional axes may serve as potential molecule biomarkers for diagnosis or therapeutic strategy (189).

## Future Directions

Further studies are needed to identify more possible sources of IL-22 and the pathways involved in its signaling. Furthermore, role of epigenetic modifications, transcription factors, non-coding RNAs in IL-22 regulation needs to be further studied. Understanding IL-22 biology for the potential development of vaccines needs to be further researched to utilize IL-22 for vaccine development. The factors and conditions that control the protective and pathological role of IL-22 needs to be studied further. Owing to its therapeutic potential in inflammatory diseases like psoriasis, ulcerative colitis, liver and pancreatic damage, graft-versus-host disease, certain infections and tumors, and its association with disease staging, IL-22 can prove to be useful for therapeutic and prognostic purposes in the future.

## Author Contributions

SM and VL conceived the idea, designed the lay out, and critically reviewed the manuscript. SM, TA, FM, and RP reviewed the literature and drafted the manuscript. All authors approved the final draft.

## Conflict of Interest

The authors declare that the research was conducted in the absence of any commercial or financial relationships that could be construed as a potential conflict of interest.
